# Platelet activating factor-induced expression of p21 is correlated with histone acetylation

**DOI:** 10.1038/srep41959

**Published:** 2017-02-03

**Authors:** Elisabetta Damiani, Nahum Puebla-Osorio, Bree M. Lege, Jingwei Liu, Sattva S. Neelapu, Stephen E. Ullrich

**Affiliations:** 1 Dipartimento di Scienze della Vita e dell’Ambiente, Universita’ Politecnica delle Marche, Ancona, Italy; 2 Department of Lymphoma and Myeloma, The University of Texas, MD Anderson Cancer Center, Houston, Texas, USA; 3 Department of Immunology and The Center for Cancer Immunology Research, The University of Texas MD Anderson Cancer Center, Houston, Texas, USA; 4 The University of Texas Graduate School for Biomedical Sciences at Houston, The University of Texas, MD Anderson Cancer Center, Houston, Texas, USA

## Abstract

Ultraviolet (UV)-irradiated keratinocytes secrete the lipid mediator of inflammation, platelet-activating factor (PAF). PAF plays an essential role in UV-induced immune suppression and skin cancer induction. Dermal mast cell migration from the skin to the draining lymph nodes plays a prominent role in activating systemic immune suppression. UV-induced PAF activates mast cell migration by up-regulating mast cell CXCR4 surface expression. Recent findings indicate that PAF up-regulates CXCR4 expression via histone acetylation. UV-induced PAF also activates cell cycle arrest and disrupts DNA repair, in part by increasing p21 expression. Do epigenetic alterations play a role in p21 up-regulation? Here we show that PAF increases Acetyl-CREB-binding protein (CBP/p300) histone acetyltransferase expression in a time and dose-dependent fashion. Partial deletion of the HAT domain in the CBP gene, blocked these effects. Chromatin immunoprecipitation assays indicated that PAF-treatment activated the acetylation of the p21 promoter. PAF-treatment had no effect on other acetylating enzymes (GCN5L2, PCAF) indicating it is not a global activator of histone acetylation. This study provides further evidence that PAF activates epigenetic mechanisms to affect important cellular processes, and we suggest this bioactive lipid can serve as a link between the environment and the epigenome.

The ultraviolet (UV) radiation in sunlight is the principal cause of both melanoma and non-melanoma skin cancer. Although most of the energy contained with UV radiation is absorbed within the very top layers of the skin, exposure to UV induces systemic immune suppression, which has been identified as a major risk factor for skin cancer induction^1^. One of the early steps in the process leading to the induction of immune suppression is the release of platelet activating factor (PAF; 1-*O*-alkyl-2-acetyl-sn-glycero-3-phosphocholine), by UV-irradiated keratinocytes^2^,^3^. We and others have provided evidence indicating that PAF plays an essential role in both UV-induced immune suppression and skin cancer induction^4^,^5^,^6^,^7^,^8^,^9^. Dermal mast cells are targeted by keratinocyte-derived PAF. Mast cells are essential for the development of immune suppression, as mast cell-deficient mice are resistant to UV-induced immunosuppression^10^. Dermal mast cell prevalence is also associated with UV-induced skin cancer induction^11^,^12^. An important step in the process leading to systemic immune suppression involves mast cell migration from the skin to the draining lymph nodes^13^, where they secrete the immune regulatory cytokine interleukin (IL)-10 and block T cell function and antibody formation *in vivo*^14^. Mast cell migration is driven by PAF-induced up-regulation of the C-X-C chemokine receptor type 4 (CXCR4) on mast cells^15^. Immune suppression and skin cancer induction is absent in UVB (290–320 nm)-irradiated mice treated with a CXCR4 antagonist^13^,^16^. Interestingly, PAF-induced CXCR4 expression is associated with increased histone acetylation on the promoter of this gene^17^.

The carcinogenic effect of UV radiation results from its ability to induce DNA mutations in tumor suppressor genes^18^,^19^ and also to modulate essential cellular processes such as DNA repair and immune suppression. UV-induced PAF contributes to these effects in that blocking the binding of PAF to its receptor blocks the induction and progression of UV-induced skin cancer and DNA repair^7^,^8^. We recently provided data indicating that PAF up-regulates the expression of the cyclin-dependent kinase (CDK) inhibitor p21^CDKN1A^ and disrupts DNA repair^20^. p21 is a well-studied, potent inhibitor that binds to, and inhibits the activity of several cyclins and CDK complexes. It belongs to the CIP/KIP family of cell cycle regulators and is implicated in many important and diverse regulatory functions of fundamental biological processes^21^. First and foremost it functions as a regulator of cell cycle progression, especially at the G1/S checkpoint^22^. Consequently, it plays a pivotal role in cell quiescence, senescence and differentiation^23^. p21 also participates in other regulatory roles such as in p53-dependent and/or independent apoptosis and in transcriptional regulation, either positively or negatively^24^,^25^. Additionally, p21 activity appears to be necessary for DNA repair in the presence of low levels of genotoxic stress, while it is degraded in the presence of extensive DNA damage to favor apoptotic cell death^26^. A more recently described function of p21 is its involvement in cell motility. In tumor cells, high levels of p21 favor Rho kinase inhibition with consequent enhanced cell movement thus contributing to tumor metastasis and invasion^27^. Some studies have shown that p21 may favor cell survival and proliferation with a consequent oncogenic potential^28^. Furthermore, it appears that p21 is essential for cell migration induced by the inflammatory cytokine IL-20^29^.

Histone modification is commonly associated with transcriptional regulation of gene expression^30^. The profound effect PAF has on histone acetylation leading to CXCR4 expression in human mast cells^17^ led us to question whether PAF modulated other genes at the epigenetic level. We were particularly interested in furthering our understanding of how PAF affects the regulation of p21. Given that p21 expression is usually regulated at the transcriptional level^31^,^32^, and that one of the main events regulating transcriptional activation is histone acetylation, we decided to focus our attention on the possible effects of PAF on acetylation of p21.

## Results

### PAF activates p21 in mast cells via an epigenetic mechanism

**Figure 1:**
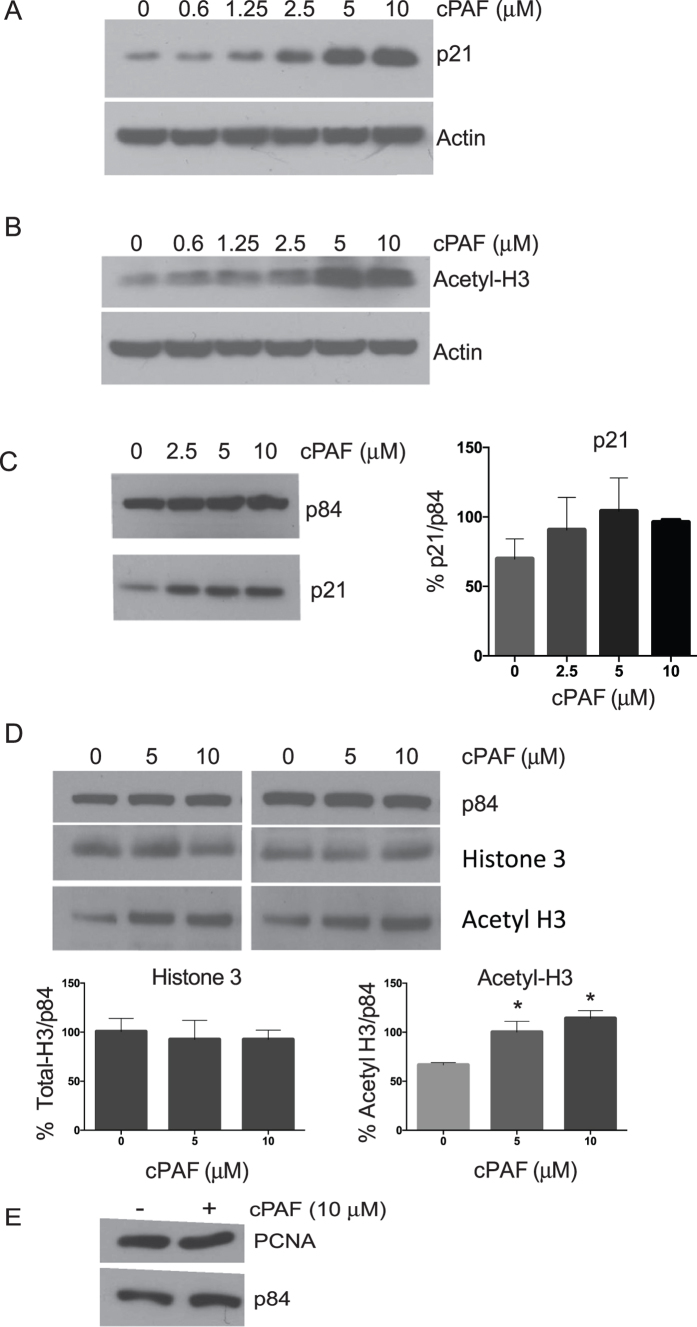
Platelet-activating factor increases the expression of p21 and acetylated histone-H3. Protein expression in HMC-1 cells was analyzed by immunoblotting. (**A**) p21 expression after 24 h incubation with different concentrations of cPAF, β-actin is the loading control. (**B**) Acetyl-H3 expression in cells treated with different concentrations of cPAF for 24 h, β-actin is the loading control. (**C**) p21 expression in normal mast cells harvested 24 h after cPAF treatment, and its corresponding quantification using NIH ImageJ software, p84 is the loading control. (**D**) Total H3 and acetyl-H3 expression in mast cells treated with 5 and 10 μM cPAF, and its corresponding quantification, p84 is the loading control, *Indicates p < 0.05; One-way ANOVA N = 3. (**E**) PCNA expression after 24 h incubation with 10 μM cPAF.

**Figure 2:**
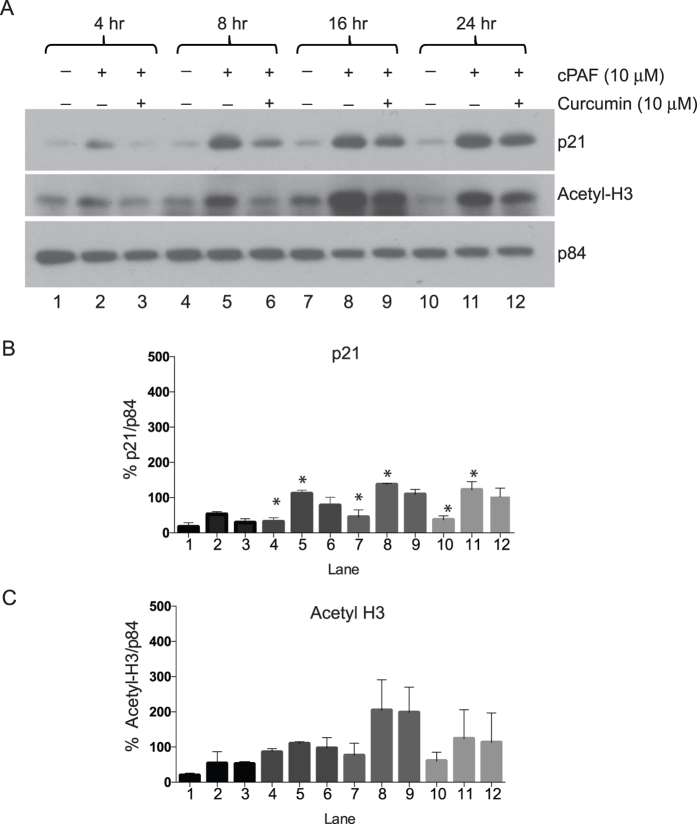
Time course for increased expression of p21 and acetylated histone-H3 by platelet-activating factor. (**A**) Time course of cPAF (10 μM)-induced p21 and acetyl-H3 expression and the effect of 10 μM curcumin at each time point was analyzed by immunoblotting, p84 is the loading control. (**B**) Quantification of p21 expression and (**C**) Acetyl-H3 expression using NIH ImageJ software. *Indicates p < 0.05; One-way ANOVA, N = 3. Lane numbers correspond to the lanes of the immunoblot presented in panel A.

We observed previously that treating mast cells with carbamyl PAF (cPAF), a non-hydrolyzable bioactive analogue of PAF, provokes a myriad of changes in cell cycle regulation in HMC-1 cells, as well as in normal mast cells^17^,^20^. These include the deregulation of the G2M regulatory complex through the disruption of cyclin B1 and CDK2, which lead to mitotic catastrophe. We also demonstrated that cPAF induced a robust activation of p21 that contributed to cell cycle arrest^20^. Moreover, PAF up-regulates mast cell surface expression of CXCR4 by activating histone acetylation^17^. Previously we noted that treating either HMC-1 cells or normal mast cells with cPAF suppressed the expression of DNA methyltransferases (DNMT) 1 and 3b, and we observed no difference in the methylation status of CpG islands in the promoter region of cPAF-treated mast cells^17^. Thus, our approach was to determine whether histone acetylation affected the cPAF-induced activation of p21, and whether blocking histone acetylation *in vitro* suppressed the cPAF-induced expression of p21. Mast cell p21 expression progressively increases when cells are treated with increasing concentrations of cPAF (Fig. 1A). Similarly, we noted that cPAF treatment resulted in a dose-dependent increase in histone H3 acetylation (Fig. 1B). In addition, treating normal mast cells with cPAF had an identical effect; p21 expression increased in a dose dependent manner (Fig. 1C). We also assessed the effect of cPAF on the expression of total H3, and noted a negligible influence on this histone as compared to its effect on acetylated H3 (Fig. 1D). We also noted no effect of cPAF on the expression of the cell-cycle regulator PCNA (Fig. 1E); this is in contrast to the marginal decrease in PCNA expression we reported previously^20^. Because the effect of cPAF on PCNA expression appears to be minimal and inconsistent, we decided not to pursue this further in this study. We decided to use 10 μM of cPAF in all subsequent experiments, which is in line with our previous studies and those of others^4^,^17^,^20^,^33^. At this concentration of cPAF we noted significant physiological changes *in vitro*, without affecting cell viability^20^. We also assessed different approaches to inhibit acetylation in mast cells, and decided to use curcumin^34^, as a broad inhibitor for most of our experiments. Notably, curcumin decreased the cPAF-induced expression of p21 at 10 μM (Suppl. Fig. 1A, lane 4). In a time course experiment we found that 10 μM cPAF induced the expression of p21 as early as 4 h post exposure, with a steady increase up to 24 h. The addition of 10 μM curcumin depressed the cPAF-induced expression of p21 as soon as 4 h post addition (Fig. 2A and B). The inhibitory effect of curcumin on the expression of p21 was diminished at the 24 h time point (Fig. 2A and B). To corroborate the effect of curcumin on acetylation, we analyzed its influence on acetylated H3 (H3K9/14/18/23/27), and observed a decrease in the cPAF-induced histone acetylation at the same time points. The effect on H3-acetylation was most pronounced 4 and 8 h post treatment, and diminished at the later time points (Fig. 2A and C).

### PAF activates acetylation

**Figure 3:**
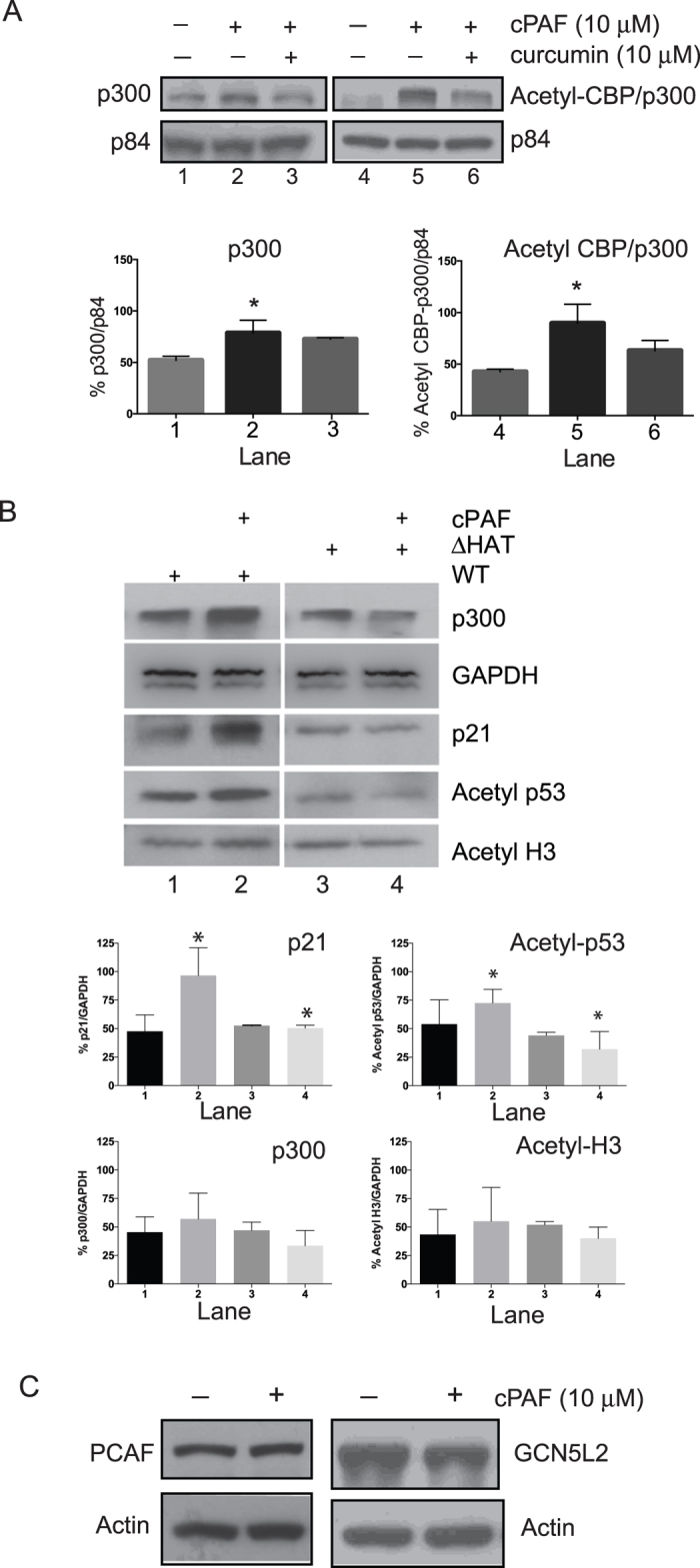
Platelet-activating factor increases the expression of p300 and acetylated CPB/p300. Protein expression in HMC-1 cells was analyzed by immunoblotting. (**A**) p300 and Acetyl-CBP/p300 expression after 24 h incubation with 10 μM cPAF with or without 10 μM curcumin, and the corresponding protein quantification using NIH ImageJ software, p84 is the loading control. (**B**) Protein expression in wild-type (lanes 1 and 2) or mutant (HAT deletion, lanes 3 and 4) OCI-Ly7 cells exposed to cPAF; acetyl p53 is the control target to asses acetylation. All proteins analyzed, p300, p21, acetyl-p53 and acetyl-H3 were quantified using NIH ImageJ software, *Indicates p < 0.05; One-way ANOVA N = 3. (**D**) No effect of cPAF on PCAF or GCN5L2 expression, β-actin is the loading control.

Note however, that although curcumin treatment depressed PAF-induced up regulation of p21, Acetyl-H3 (Fig. 2) and Acetyl-CBP/p300 (Fig. 3A) expression, the effect did not always reach statistical significance. The problem with using curcumin, indeed the problem associated with the use of any pharmacological inhibitor (specificity, selectivity, off target side effects) could explain these results. To directly address this concern and to further assess the effect of cPAF on the acetylation of p21, we used a genetically engineered lymphoma line cell, OCI-Ly7, which was constructed with a partial deletion in the HAT domain on CBP (See Material and Methods). This deletion is known to reduce acetyl transferase function^35^. To test our model we included another target of CBP, acetyl p53, and we exposed both parent (WT) and mutant (ΔHAT) OCI-Ly7 cells to 10 μM of cPAF for 24 h. Unlike mast cells, cPAF-treated wild type OCI-Ly7 cells did not significantly up-regulate p300 expression, whereas the expression of p21 and acetyl p53 was significantly up-regulated (Fig. 3B, lanes 1 and 2). The mutant cells carrying a partial deletion of the HAT domain show a significant reduction of PAF-induced acetyl-p53 (Fig. 3B, lane 3 and 4), as compared to the WT control (Fig. 3B, lanes 1 and 2). Moreover, cPAF-induced expression of p21 was significantly suppressed in the cells containing a partial deletion of the HAT domain (Fig. 3B, lanes 3 and 4). We saw at best a modest cPAF-induced expression of acetyl-H3 between the mutant and the WT lymphoma cells (Fig. 3B). Unlike what we found for p300 and acetyl-CBP/p300, PAF treatment did not result in increased protein expression of GCN5L2 and PCAF (Fig. 3C). In view of the fact that PAF-induced up-regulation of p21 was absent in cells engineered to have a reduced acetyl transferase activity, these data strongly suggest that cPAF induces the expression of p21 through acetylation-induced transcriptional activation.

### PAF-induced acetylation of the p21 promoter

**Figure 4:**
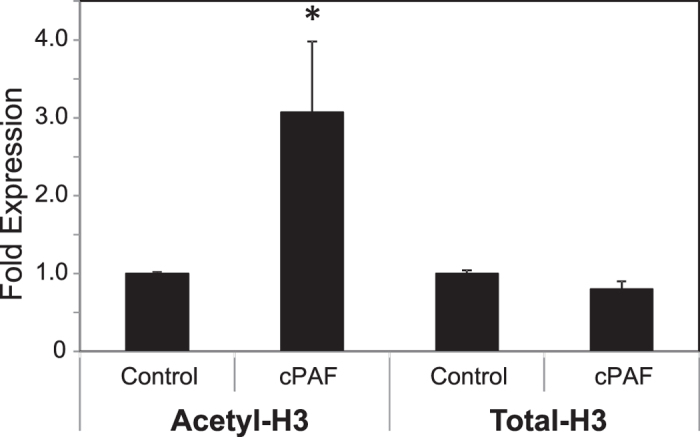
Effect of platelet-activating factor on the promoter region of the p21 gene. Acetylation of the promoter region of p21 in HMC-1 cells was analyzed using ChIP followed by qPCR. The fold expression of acetylated histone-H3 associated with the p21 gene promoter was analyzed in cells treated with 10 μM cPAF and harvested 24 hours later. Total-H3 was used as positive control and data were normalized against input DNA. Data represent the mean ± SEM (N = 3). *p < 0.05 vs. control (Mann-Whitney *U*-test).

To confirm that the observed increase in acetylated-H3 protein expression correlates with the acetylation status of the p21 promoter, we performed a ChIP assay on cPAF-treated and control samples using an antibody for H3 (H3K9/14/18/23/27), which detects acetylated histone H3. We analyzed the resulting DNA by quantitative PCR using specific primers covering the promoter region of the human p21 gene. We found that cPAF induced approximately a three-fold increase in acetylated-H3 on the promoter region of p21 (Fig. 4). These findings provide direct evidence that cPAF up-regulates histone acetylation of the p21 promoter region.

## Discussion

Acetylation of lysine residues on histones is one of several post-translational modifications of nucleosomes and is usually associated with an open chromatin structure allowing accessibility of nucleosomal DNA for transcription^30^. It is a reversible process: histone acetyltransferases (HATs) catalyze the covalent transfer of the acetyl group from acetyl-CoA to the epsilon amine group of lysine side chains leading to loss in the positive charge, while histone deacetylases (HDACs) re-establish the positive charge by removing the acetyl groups, thereby facilitating a closed chromatin structure and hence transcriptional repression^36^,^37^.

The work presented here focused on p21 induction by cPAF and the possible correlation with epigenetic modulation via histone acetylation. This hypothesis appeared reasonable given our recent findings of cPAF-induced CXCR4 promoter acetylation and the literature linking transcriptional activation of p21 through histone acetylation^38^,^39^,^40^,^41^,^42^. Many studies have shown that HDAC inhibitors (HDACi) strongly activate the expression of p21 through the Sp1/Sp3 sites on the p21 promoter, and that there is enhanced histone acetylation around the p21 promoter, suggesting that acetylation is the major mechanism for regulation of the p21 gene in several transformed cell lines^43^,^44^. The de-repression of p21 by HDACi subsequently results in growth inhibition and differentiation of cancer cells. By exploiting this property several drugs have been developed and have demonstrated therapeutic potential in a variety of malignancies, including T-cell lymphomas and solid tumors^45^. For example, the hydroxamic acids, Trichostatin A and Vorinostat have been reported to effectively induce p21 expression, cell cycle arrest and apoptosis in human gastric, oral and bladder cancer. This effect appears to be due mainly to the enhancement of acetylation of the histones H3 and H4 around the promoter region^46^. In this regard, our results suggest that cPAF appears to share similarities in its action with these HDACi, since treatment of mast cells with cPAF leads to increased p21 protein expression, which is concomitant with increased protein expression of acetylated histone H3.

Further evidence for the involvement of histone acetylation in cPAF-induced p21 expression came from studying protein expression of the HAT acetyl-CBP/p300, which was up regulated after cPAF treatment. More importantly, we demonstrated that cPAF-induced expression of p21 is absent in OCI-Ly7 cells that have impaired acetyl transferase activity. CBP and p300 are well-known functionally related co-activators and integrators for signal transduction and are involved in a variety of cellular signaling pathways ranging from calcium signaling, response to hypoxia, Notch signaling, and NF-κB signaling. They contain multiple modules for protein-protein interactions and can serve as adaptors for transcriptional assembly and recruitment. Importantly, they are endowed with intrinsic and extrinsic histone/protein acetyltransferase activity releasing repression of transcription activation^47^,^48^. The auto-acetylation of p300/CBP has been shown to stimulate its acetyltransferase activity and is the result of an efficient and cooperative intermolecular reaction on a regulatory loop within the acetyltransferase domain^49^. CPB/p300 is known to be a co-regulator of p21 and is linked to the expression of the p21 promoter after different stimuli^40^,^43^,^50^. It is necessary for p21-dependent differentiation of keratinocytes^51^ and p21-dependent cell cycle arrest and differentiation of muscle cells induced by MyoD^52^. In conjunction with Sp1, CBP/p300 also appears to be involved in progesterone induction of the p21 promoter in HeLa cells^43^. Because p300/CBP, Sp1/Sp3 and GC-box are tightly interrelated in the promoter activity of p21^40^, it is possible that cPAF-induced promoter activation is through this link. Furthermore, knowing that hyperacetylation of histones H3 and H4 of the Sp1/Sp3 binding sites on the p21 promoter induces p21 expression^38^,^39^, coupled with our data showing that cPAF-treatment leads to p21 promoter gene acetylation and protein expression, strongly suggests that this might be a mechanism worth pursuing in the future. Likewise investigating the possible transcription factors induced by cPAF that could co-localize on the promoter of p21 may have merit.

Previously we showed that cPAF-induced up regulation of p21 occurred through a p53-dependent mechanism^20^. The inhibition of p53 acetylation in mutant OCI-Ly7 cells that have impaired acetyl transferase activity suggests an alternative explanation for the inhibition of p21 expression we report here. It is conceivable that cPAF-induced acetylation of p53 is responsible for up-regulating p21, which is absent in a cell line with reduced acetyl transferase function. Regardless of the exact mechanism involved, the inability of cPAF to up regulate the expression of p21 in mutant OCI-Ly cells indicates that histone acetylation is essential.

A recent report indicates that p21 was over-expressed in epidermal Langerhans cells (LCs) following treatment with ionizing radiation, and that it was a key modulator of the resistance of LCs to radiation. Moreover, LCs migrated to the skin draining lymph (LNs) in a CCR-7 dependent manner that leads to an increase in tumor infiltrating T regulatory cells and resistance to radiotherapy^53^. Part of this response involves an immune inhibitory program that is permissive for the growth of skin cancer. In fact, UV-irradiated LCs also promote UV radiation-induced immune suppression^54^,^55^. Dermal mast cells also migrate to the LNs upon UV exposure and this is a key step in UV-induced suppression that is mediated by keratinocyte-derived PAF^15^. In mast cells, this migration is driven by PAF-induced over expression of CXCR4, which is linked to hyperacetylation of its promoter^17^. However, as shown in the present study, PAF also induces over expression of p21 and hyperacetylation of its promoter. Because p21 up-regulation has been shown to promote LC migration to LNs following an external stimulus^53^, it is reasonable to ask whether migration of mast cells is also dependent, in part on p21 up-regulation and whether chromatin modifications also underlie the migration of LCs to the draining lymph nodes. It would appear that both type of innate immune cells, LCs and mast cells, might share common pathways leading to systemic immune suppression and that an epigenetic dimension could be operative in both cases. In this regard is important to note that one report in the literature links PAF to LC migration^56^.

It is worth mentioning that our study is an *in vitro* study, and as mentioned earlier, the concentrations of cPAF used are in line with reports in the literature where cPAF was used to activate cells *in vitro*. The tissue concentrations of PAF found *in vivo* are in the picomolar range^2^ but under certain conditions such as inflammation and cancer serum levels of PAF, similar to those used here (10^−7^ molar), have been reported^57^,^58^. Also, PAF in the serum has a limited half-life (3–13 minutes) due to the action of PAF acetyl-hydrolase^59^. Moreover platelets and endothelial cells are known to produce PAF but do not secrete it^60^. This indicates that the cell-associated form of PAF is active, and suggests that local concentration of PAF may be very high in inflamed tissues. For these reasons, we suspect that it is possible that PAF *in vivo* may be exerting its multiple biological effects in part, by affecting chromatin modification.

Overall, this concise study provides further evidence for the epigenetic effects of PAF on yet another gene, p21, which is an important key protein for many different cell functions and regulatory pathways^21^. PAF has been shown to act as a unique biological regulator in a variety of physiological and pathological processes in many cell types and tissues^61^,^62^. The results described here, together with those previously reported^17^,^20^ raises far-reaching questions on the implications that PAF may have on cell cycle, DNA damage response, and immune function, as it appears at least in part to exert its effects via chromatin modifications. In this regard it is important to note that PAF is not a global activator of histone acetylation and cell cycle regulators. It had no effect on other acetylating enzymes such as GCN5L2 and PCAF and other cell cycle regulators like PCNA. In the future, it will be important to extend our understanding of the function of PAF and acetylation to obtain an increasingly clearer view of the molecular events that occur during PAF-induced transcriptional activation.

## Materials and Methods

### Reagents

Carbamyl PAF (cPAF), a non-hydrolyzable bioactive analogue of PAF was obtained from Enzo Life Sciences (Farmingdale, NY). cPAF was prepared as a 10 mM stock solution in water. Curcumin was purchased from Sigma-Aldrich (St Louis, MO) and was prepared as a 35 mM stock solution in DMSO. Both were aliquoted and stored at −20 °C until use. Antibodies specific for p300 (sc-585) and p21 (sc-397) were purchased from Santa Cruz (Dallas, TX). Anti-acetyl-H3 (ab47915) and anti-total-H3 (ab1791) were acquired from Abcam (Cambridge, MA). Antibodies specific for p84 (GTX70220) and GAPDH (GTX627408) were from GeneTex (Kennesaw, GA) and those for β-actin (A2228) were from Sigma-Aldrich Chemical Co. Anti-mouse (7076 S), anti-rabbit (7074 S) HPR antibodies, anti-Acetyl-CBP/p300 (4771 S), anti-GCN5L2 (3305 P), anti-PCAF (3378 S), and anti-Acetyl-p53 (2525 S) were from Cell Signaling Technology (Danvers, MA). Anti-PCNA antibody was from Dako (Carpinteria, CA). All other analytical grade chemicals were purchased from Sigma Aldrich.

### Cell culture

The human transformed mast cell line HMC-1 was kindly provided by Dr. J. H. Butterfield, Mayo Clinic, Rochester, MN^63^. The cell lines used here were validated by STR DNA fingerprinting by the MD Anderson Cancer Center Characterized Cell Line Core using the AmpFℓSTR identifier kit according to manufacturer’s instructions (Applied Biosystems, Thermo Scientific, Rockford, IL). The STR profiles were compared to known ATCC fingerprints (ATCC.org), to the Cell Line Integrated Molecular Authentication database (CLIMA) version 0.1.200808 (http://bioinformatics.istge.it/clima/), and to the MD Anderson fingerprint database. The STR profiles matched known DNA fingerprints or were unique. Cells were cultured in complete RPMI-1640 medium containing 10% heat inactivated fetal calf serum (Thermo Scientific, Rockford, IL), under standard culture conditions (37 °C, 5% CO_2,_ humidified atmosphere) and passaged every 3–4 days. Prior to each experiment, HMC-1 cells were seeded at a density of 5 × 10^5^ cells/ml in 60 × 15 mm petri dishes and treated with cPAF and/or curcumin for various time points (6–24 h). Control samples lacked cPAF and curcumin. The cells were then harvested accordingly for immunoblotting, chromatin immunoprecipitation (ChIP) assay, and quantitative polymerase chain reaction (qPCR) analysis. Normal mast cells were derived from a buffy coat obtained from an undisclosed healthy donor from the Gulf Coast Regional Blood Center (MDACC IRB LAB-030-390) as described previously^17^,^20^. CD34+ cells were cultured in complete RPMI-1640 medium containing 10% heat inactivated fetal calf serum supplemented with human IL-6, IL-3 and Stem Cell Factor. Four to 6 weeks later all the viable cells stained positive for CD117 (cKit), tryptase, and toluidine blue.

### Immunoblotting

For protein extraction, cell pellets were lysed in 150 μl RIPA buffer and the lysates stored at −80 °C. Upon thawing, protein concentration was determined (Pierce BCA Protein Assay Kit, Thermo Scientific) and samples containing equal amounts of protein were loaded in each well and separated on 8% or 12% SDS-PAGE gels. Protein transfer was performed overnight at 4 °C on PVDF membranes and then probed for the targets of interest as reported previously^17^. Each membrane was cut along the approximate molecular weight of each protein of interest; this is to take advantage of each run and to be able to detect more than one target at once. The antibodies used include the following: anti-p21 (1:500, Santa Cruz), Acetyl-H3 (1:1500, Abcam), PCNA (1:1500), p300 (1:1000), Acetyl-CBP/p300 (1:1000), PCAF (1:500), GCNL52 (1:500), the loading controls p84 (1:2500), β-actin (1:5000), GAPDH (1:1500), Acetyl p53 (K382, 1:1000), and the secondary anti-mouse (1:4000) and anti-rabbit (1:3000) HRP-labeled antibodies. Protein bands were detected using an enhanced chemiluminescent substrate (Supersignal West Dura, Thermo Scientific) and captured on X-ray films. Quantification of protein bands was carried out with NIH ImageJ software (http://rsb.info.nih.gov/nih-image/). The data are reported as the percentage of intensity of the experimental protein band compared to the intensity of the control band (i.e. β-actin or p84).

#### Targeted Deletion of the CBP HAT Domain

Two separate gRNAs (gRNA1 target sequence: 5-ctgtgcGgaggcaacgtggccgG-3, gRNA2 target sequence: 5-aaagagcttgctacgtgcccagG-3) expression vectors R1 and R2, were constructed using pSpCas9(BB)-2A-Puro (PX459) V2.0. PX459 was a gift from Feng Zhang (Addgene plasmid #62988)^64^. R1 and R2 work together to delete 254 bp from the CBP genomic DNA (NG_009873, 141494-141747 region, the exon that includes P1442-L1464, which is a part of HAT domain). R1 and R2 were electroporated (Neon® Transfection System, Invitrogen, USA) into B-cell non-Hodgkin lymphoma OCI-Ly7 cells, which do not contain known^65^,^66^ mutations on the CBP gene. After selection with puromycin for 2 days, a single clone was isolated using serial dilution in a 96 well plate. Two weeks later, genomic DNA of each single clone was extracted and genotyping was performed using PCR (F-R1446-V: 5-ttgcagcctgaatgacagagc-3,R-R1446-V: 5-atgttctgaaactgacttgtgata-3). R4, R10, R12, R14 and R16 are homologous deletion clones, which are about 254 bp shorter than wild type Ly7 cells. PCR product of R4, R10, R12, R14 and R16 was purified and sent for Sanger sequencing using the same primers.

### ChIP and qPCR analysis

Chromatin immunoprecipitation was performed using a ChIP assay kit (17–295, EMD Millipore Co., Temecula, CA) according to the kit’s protocol and as outlined previously^17^. The purified DNA obtained was then subjected to quantitative polymerase chain reaction (qPCR) analysis with appropriate primer pairs for human p21 promoter region, which spanned from 2750 to 2834 bp: 5′-TGGCTCTGATTGGCTTTCTGG-3′ (forward) and 5′-GGCAGCCCAAGGACAAAATAG-3′ (reverse). The primer sequences used were blasted against the human genome database, which generated 100% alignment with the promoter region of p21. The samples were run and analysed by performing qPCR using the input samples for normalization. All samples were diluted five times and 3 μl of these dilutions were used as template in qPCR reactions (total volume 15 μl). The reactions were run on a CFX96 Real-Time PCR Detection System (Bio-Rad) under the following PCR conditions: 95 °C for 2 min, 40 cycles at 95 °C for 5 sec, and 60 °C for 30 sec. The results obtained were analysed using the CFX Manager Software (Bio-Rad). The cycle threshold (C_t_) method using the 2^−ΔΔCt^ formula as described by Arya and colleagues^67^ was used to measure the fold changes in expression level in cPAF treated vs control samples of acetylated histone-H3 in the promoter region of p21.

### Statistical Analysis

Each experiment was repeated at least three times. Statistical differences between the control and cPAF-treated samples were analysed using the Mann-Whitney U test or a One-way ANOVA. Significant differences were defined as p < 0.05.

## Additional Information

**How to cite this article**: Damiani, E. *et al*. Platelet activating factor-induced expression of p21 is correlated with histone acetylation. *Sci. Rep.* **7**, 41959; doi: 10.1038/srep41959 (2017).

**Publisher's note:** Springer Nature remains neutral with regard to jurisdictional claims in published maps and institutional affiliations.

## Supplementary Material

Supplementary Information
