# Supplementary material for: Platelet activating factor-induced expression of p21 is correlated with histone acetylation
Source: Sci Rep. 2017 Feb 3;7:41959. doi: 10.1038/srep41959 (PMC5291204; doi:10.1038/srep41959)
Supplement: Supplementary Information [file srep41959-s1.pdf]

Platelet activating factor-induced expression of p21 is correlated with histone acetylation

Elisabetta Damiani, Nahum Puebla-Osorio, Bree M. Lege , Jingwei Liu , Sattva S. Neelapu,
Stephen E. Ullrich

Supplemental Figure 1

A

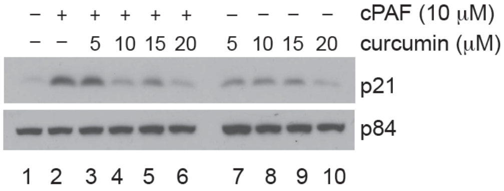

Supplemental Figure 1. Curcumin prevents the cPAF-induced expression of p21. (a) p21 expression in cells after 16 h incubation in the presence of different concentrations of curcumin, either in the presence or absence of 10 μM cPAF, p84 is the loading control.

Supplemental Figure 2

A

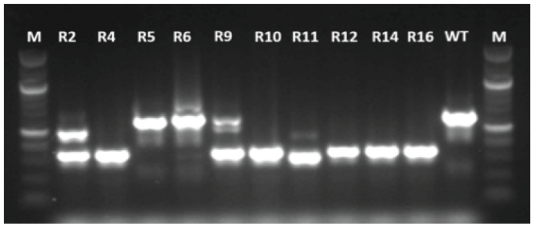

B

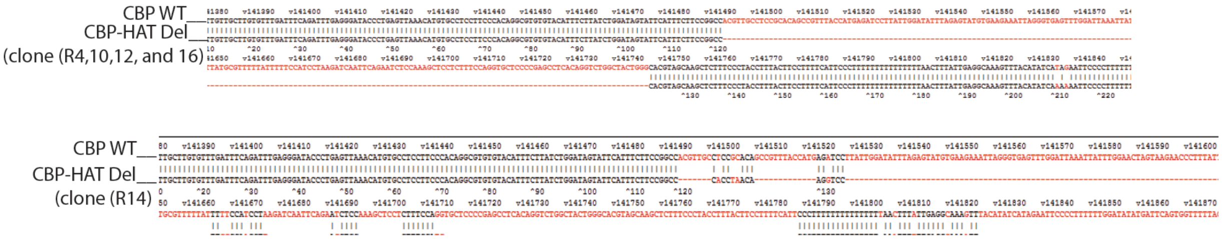

Supplemental Figure 2. (a) Genotyping results of single clones after the HAT deletion in OCI-Ly7 cells: M, 100bp DNA ladder; WT, wild type control, R4, R10, R12, R14, and R16 are clones with the HAT deletion. (b) The deleted region of each single clone was verified by Snager sequencing.
